# Predicting dispersal of auto-gyrating fruit in tropical trees: a case study from the Dipterocarpaceae

**DOI:** 10.1002/ece3.1469

**Published:** 2015-04-02

**Authors:** James R Smith, Robert Bagchi, Judith Ellens, Chris J Kettle, David F R P Burslem, Colin R Maycock, Eyen Khoo, Jaboury Ghazoul

**Affiliations:** 1 Ecosystem Management, Department of Environmental Systems Science, ETH ZurichUniversitaetstrasse 16, 8092, Zurich, Switzerland; 2 Department of Ecology and Evolutionary Biology, University of Connecticut75 N. Eagleville Road, Unit 3043, Storrs, Connecticut, 06269, USA; 3 Eaternity, Viaduktstrasse 93-958005, Zürich, Switzerland; 4 Institute of Biological and Environmental Sciences, University of AberdeenCruickshank Building, St Machar Drive, Aberdeen, AB24 3UU, UK; 5 School of International Tropical Forestry, Universiti Malaysia SabahSabah, Malaysia; 6 Sabah Forestry Department, Forest Research CentreSabah, Malaysia

**Keywords:** Auto-gyrating fruit, Borneo, Dipterocarpaceae, inverse wing loading (IWL), seed dispersal, tropical forest

## Abstract

Seed dispersal governs the distribution of plant propagules in the landscape and hence forms the template on which density-dependent processes act. Dispersal is therefore a vital component of many species coexistence and forest dynamics models and is of applied value in understanding forest regeneration. Research on the processes that facilitate forest regeneration and restoration is given further weight in the context of widespread loss and degradation of tropical forests, and provides impetus to improve estimates of seed dispersal for tropical forest trees. South-East Asian lowland rainforests, which have been subject to severe degradation, are dominated by trees of the Dipterocarpaceae family which constitute over 40% of forest biomass. Dipterocarp dispersal is generally considered to be poor given their large, gyration-dispersed fruits. However, there is wide variability in fruit size and morphology which we hypothesize mechanistically underpins dispersal potential through the lift provided to seeds mediated by the wings. We explored experimentally how the ratio of fruit wing area to mass (“inverse wing loading,” IWL) explains variation in seed dispersal kernels among 13 dipterocarp species by releasing fruit from a canopy tower. Horizontal seed dispersal distances increased with IWL, especially at high wind speeds. Seed dispersal of all species was predominantly local, with 90% of seed dispersing <10 m, although maximum dispersal distances varied widely among species. We present a generic seed dispersal model for dipterocarps based on attributes of seed morphology and provide modeled seed dispersal kernels for all dipterocarp species with IWLs of 1–50, representing 75% of species in Borneo.

## Introduction

Seed dispersal represents the primary, and often sole, opportunity for seed-bearing plants to colonize new habitats and overcome the constraints to the survival of progeny close to conspecific adults. Differential seed dispersal among species contributes to plant community structure and dynamics by determining which species or combination of species reach suitable establishment sites. Trade-offs among traits governing dispersal, establishment, and survival mean that dispersal can have long-term implications for plant community structure (Rees et al. 2001). Furthermore, seed dispersal is an integral process involved in several mechanisms of species coexistence (Chesson 2000), including neutral theory (Hubbell 2001), distance- or density dependence (Janzen 1970; Connell 1971), and the competition–colonization trade-off (Tilman 1994). Seed dispersal is similarly critical to species persistence, as the negative effects of small population size may be ameliorated by dispersal capability through the formation of more resilient metapopulations (Hanski et al. 2013) and by maintaining gene flow among populations. Long distance dispersal capability determines the rate of population spread into favorable habitat, for example, postglacial range expansion or climate-driven range shifts, and of founding events in new locations such as oceanic islands.

Understanding seed dispersal is therefore of fundamental importance to plant ecology, and seed dispersal capability is consequently included in a range of dynamic models of plant ecological processes. Despite this central role, there remains a dearth of accurate dispersal kernels for the majority of plant species, and those available are often for species from temperate grasslands or temperate forests. Many models that include a seed dispersal component, including those that address climate-driven range shifts, species coexistence, spatial aggregation patterns, and habitat connectivity, are limited to generalizing dispersal capacity across many species using, for example, dispersal syndromes (Seidler and Plotkin 2006; Bagchi et al. 2011). Increasing the number of taxonomic groups or fruit morphologies for which we have accurate dispersal kernels can therefore improve ecological modeling of seed dispersal and derived processes.

An understanding of seed dispersal also has applied relevance for forest management, especially in the context of anthropogenic environmental change. Of particular interest are the tropical forests of South-East Asia, which have the highest annual deforestation rates in the tropics (Sodhi et al. 2009). Much of the remaining forest cover is degraded and fragmented, with uncertain implications for the viability of remaining tree populations and associated biodiversity (Sodhi et al. 2009; Wilcove et al. 2013). Given current economic pressures, logging and forest conversion to agriculture are likely to continue (Fisher et al. 2011).

South-East Asian lowland rainforests are dominated by trees of the Dipterocarpaceae family, which generally constitute over 40% of basal area (Newbery et al. 1992; Curran and Leighton 2000; Davies et al. 2005). The dispersal of the mostly winged fruits of dipterocarps is generally considered to be poor, but there is wide variability in fruit size and morphology which might reflect species-specific differences in seed dispersal. In view of the substantial fragmentation and degradation of South-East Asian forests, a thorough understanding of dipterocarp seed dispersal could provide insights into changing patterns of regeneration, including changes to the template on which density dependence might act, and hence shifts in future species composition.

Dipterocarp fruit are composed of a nut and calyx. In most species, the sepals become elongated to form wings which cause fruits to gyrate when abscised (Suzuki and Ashton 1996). Substantial variation exists in both nut and wing size, and in wing number which varies from zero to five. Such variation suggests substantial differences in seed dispersal among dipterocarp species based on wing and nut morphology. Hereafter, we refer to seed dispersal rather than fruit dispersal as dipterocarp fruit are single seeded, and lack of a fleshy, nutritious animal-dispersed pericarp limits potential for secondary dispersal. Thus, fruit and seed dispersal is equivalent.

Green (1980) observed that the rate of descent of single-winged fruits (samaras) is proportional to the square root of fruit “wing loading,” defined as fruit mass divided by wing surface area. Dispersal distance of a falling fruit can therefore be modeled using a simple ballistic model composed of the terminal velocity of the fruit, height of release, and lateral wind speed (Nathan et al. 2011). A slower rate of descent increases the time available for fruit to be dispersed horizontally (Green 1980). An inverse relationship is therefore expected between wing loading and dispersal distance (Augspurger and Franson 1987; Osada et al. 2001). Wing loading values can similarly be calculated for dipterocarps, and seed dispersal potential ranked on this basis (Suzuki and Ashton 1996). We hypothesize that fruit morphology mechanistically underpins dispersal distance in dipterocarps through the lift provided to seeds mediated by wing loading. We therefore hypothesize that species-level seed dispersal is correlated with wing loading. We tested this hypothesis experimentally by releasing over 650 fruit from 13 species, representing a broad range of wing loading values, from a 30-m canopy tower at a site in Malaysian Borneo to determine seed dispersal distances. Using the data generated, we constructed generic models of seed dispersal distance as a function of inverse wing loading (IWL; ratio of wing area to fruit mass) to approximate seed dispersal kernels for all dipterocarp species with IWLs of 1 to 50, which spans a range that includes 75% of all dipterocarp species found in Borneo.

## Methods

### Fruit collection

We collected mature fruit belonging to 13 dipterocarp species from three genera (10 *Shorea*, two *Dipterocarpus,* and one *Hopea*) (Table1) growing in Sepilok Forest Reserve (SFR), Malaysian Borneo (5°51′ N 117°56′ E). SFR is a 4420 ha fragment of primarily tropical lowland dipterocarp forest, ranging in altitude 0–170 m.a.s.l. (Fox 1973). Fruit were collected from the ground during the 2010 community-wide mast fruiting event from the vicinity of identified mother trees in a 160 ha inventoried plot of mature dipterocarps (diameter at breast height (d.b.h.) >30 cm). Any fruit exhibiting external signs of predation were excluded.

**Table 1:** Fruit morphology measures (± standard error) and dispersal parameters for the fruit of the 13 dipterocarp species released from the 30-m canopy tower

Species	Number of fruit	Mean Fruit dry mass (g)	Mean IWL (cm^2^/g)	Minimum distance dispersed (m)	Maximum distance dispersed (m)	Median distance dispersed (m)	90th percentile distance dispersed (m)
*Dipterocarpus humeratus*	59	19.85 (± 1.097)	9.29 (± 0.27)	0.86	10.86	3.84	7.94
*Dipterocarpus kerrii*	75	6.04 (± 0.267)	8.93 (± 0.47)	0.83	10.94	4.00	8.14
*Hopea beccariana*	38	0.16 (± 0.004)	26.39 (± 0.83)	1.30	19.90	3.96	11.26
*Shorea acuminatissima*	60	0.76 (± 0.023)	17.37 (± 0.75)	1.00	9.65	3.40	6.75
*Shorea argentifolia*	55	0.68 (± 0.022)	44.60 (± 1.86)	1.40	39.54	8.20	15.58
*Shorea beccariana*	51	3.97 (± 0.083)	21.55 (± 0.45)	0.42	17.82	6.57	12.20
*Shorea falciferoides*	61	1.64 (± 0.094)	24.85 (± 1.03)	1.11	16.33	5.20	10.61
*Shorea gibbosa*	60	1.04 (± 0.038)	11.53 (± 0.58)	0.94	7.45	2.62	3.95
*Shorea macroptera*	54	2.01 (± 0.121)	39.83 (± 1.98)	1.22	19.41	7.01	12.57
*Shorea mexistopteryx*	48	11.79 (± 0.536)	16.49 (± 0.73)	0.94	22.73	5.42	10.76
*Shorea seminis*	61	1.94 (± 0.116)	1.97 (± 0.14)	0.76	6.33	1.99	4.13
*Shorea smithiana*	61	2.64 (± 0.113)	20.95 (± 0.65)	0.86	20.71	4.84	12.00
*Shorea xanthophylla*	45	2.48 (± 0.235)	0.00 (± 0.00)	0.32	8.26	1.84	4.52

### Wing loading calculations

The air-dried mass (g) and lengths and widths of the wings (cm) were measured for each fruit. From these data, the “inverse wing loading” (IWL) was calculated, defined as “long” wing area divided by mass. Wing areas in the genera *Dipterocarpus* and *Hopea* were calculated by summing the product of wing length and wing width of their two wings. Species in the genus *Shorea* have three “long wings” and two “short wings”. We excluded short wings from the IWL calculation as their areas are much smaller than that of the long wings, and we assume they contribute little to lift (Suzuki and Ashton 1996). The wing area of *Shorea* was therefore calculated as the total area of the longest and shortest long wings multiplied by 1.5 to account for the third long wing. We use the inverse of wing loading rather than the traditional wing loading as this value generates a more intuitive dispersal index where higher values equate to higher dispersal distances. Moreover, IWL also avoids mathematical inconsistencies arising from the inclusion of fruits that lack wings (e.g., *S. xanthophylla*). Each fruit was uniquely numbered and partially covered by a thin layer of spray paint to aid recovery.

### Experimental release

Dispersal was assessed by releasing fruits individually from a 30-m canopy observation tower. The tower is located in a forest gap where no trees taller than 28 m are located within 10 m. A single fruit was released for each species in turn before repeating the cycle so as to avoid temporal autocorrelation, which might be relevant on account of changing wind speeds. Wind speed (m/sec) was recorded for the duration of each individual fruit’s flight using an electronic anemometer (Windmaster 2, Kaindl Electronic, Rohrbach, Germany) located at the release point. The maximum wind speed and mean wind speed per release were obtained from these measurements. Subsequently, the area surrounding the tower was exhaustively searched for marked fruit, and the horizontal distance travelled by each fruit was measured using a laser distance meter (Leica Disto A8, Leica Geosystems, Heerbrugg, Switzerland). The dispersal distance was measured for fruits reaching the forest floor only, as some fruits became entangled in understory vegetation. More than 90% of released fruits were, however, recovered. We assume that the dispersal from gyration encapsulates the full dispersal potential in the field, although there have been some observations of rare short-distance secondary dispersal by rodents (Maycock et al. 2005; Wells and Bagchi 2005).

### Statistical analysis

A linear mixed-effects model (LMM) was fitted to the data using the lme4 package (version 1.0.5; Bates et al. 2013) in R-3.0.2 (R Core Team, 2013). The response was measured distance dispersed, and the predictors were individual fruit IWL, wind speed during release and their interaction. Maximum wind speed was used instead of mean wind speed as this greatly improved model fit (model AIC reduced by 12 points). To control for the effect of intra- and interspecific differences in fruit morphology on dispersal, we included random effects for mother tree nested within species, nested in turn within genus. The mother tree and genus terms were subsequently dropped from the model as both accounted for <0.1% of the total variance in the response. Plus one was added to IWL and maximum wind speed to account for zeroes in these data, and subsequently, all variables were log-transformed to ensure that the residuals were normally distributed. Given difficulties in modeling the long tail of dispersal kernels (Nathan 2006), the variance was expected to increase with distance dispersed. The full model therefore initially explicitly modeled the variance as a power function of the expected mean (using the nlme package, version 3.1.113 (Pinheiro et al. 2013)). However, this model did not perform better than a simpler homoscedastic model, so the variance function was dropped for subsequent analyses.

Dispersal kernels and their 95% confidence bounds were estimated using a parametric bootstrapping approach implemented with the “bootMer” function in lme4. Approximate *P*-values for the LMM parameter estimates were calculated from the bootstrap models following Gelman and Hill (2007). Details of this bootstrap approach and approximate *P*-value calculations are provided in the supplements (Table S1). Dispersal kernels for a sample of 50 hypothetical dipterocarp species with IWLs spanning 1 to 50 were simulated using this bootstrapping technique for a range of wind speeds spanning 1 to 10 m/sec (Table S2). These IWL values represent 75% of the Dipterocarpaceae on Borneo (Data from Newman et al. (1996, 1998)). IWLs calculated using long wing area/nut volume as mass data unavailable.

## Results

Substantial variation in fruit morphology and distances dispersed were observed (Table1). Mean IWL values ranged from 0 in wingless *S. xanthophylla* to 44.60 (± 1.86) cm^2^/g in *S. argentifolia*. These species recorded the shortest and furthest dispersal distances of 0.32 m and 39.54 m, respectively. Fifty percent of all released fruits dispersed less than 4 m, and 90% were recovered within a horizontal distance of 10.5 m of the release point (Table1). The majority of fruits were released at relatively low wind speeds (mean maximum wind speed during releases was 1.72 m/sec, and the highest recorded wind speed was 10.5 m/sec). The mean maximum wind speed of 1.72 m/sec observed corresponds closely to the mean annual wind speed of 2.05 m/sec (2000–2013; data from Sandakan Airport 11 km distant, Tutiempo, 2014; http://www.tutiempo.net/en/), and therefore, conditions during the releases were close to the site’s normal atmospheric conditions.

**Figure 1:**
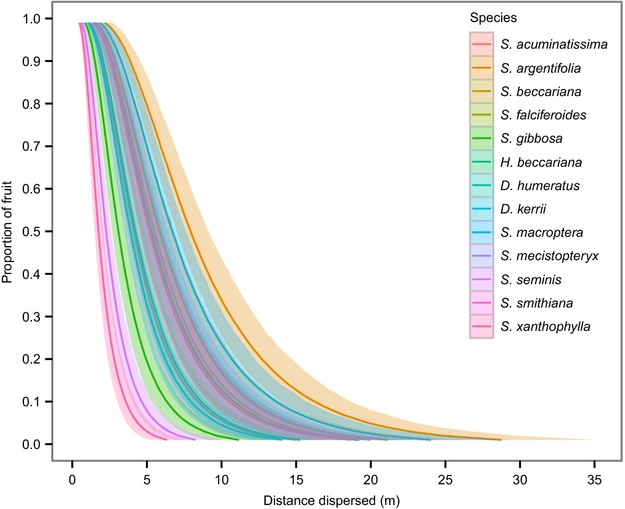
Simulated dispersal kernels of the 13 observed species released at the mean maximum wind speed of 1.72 m/sec and associated 95% confidence bands.

The best fitting LMM model included IWL, maximum wind speed, and their interaction as independent variables and species as a random effect. Significant positive effects on dispersal distance were found for IWL (*β *= 0.186, 95% C.I. = 0.0759 to 0.237, *P*-value = 0.001) and the interaction between IWL and maximum wind speed (*β *= 0.191, 95% C.I. = 0.115 to 0.259, *P*-value = 0.001), indicating that fruit disperse greater distances with higher IWLs and that this is especially true at higher wind speeds (Table2). Dispersal kernels and associated 95% confidence bands were generated for each of the 13 species released at the mean maximum wind speed of 1.72 m/sec (Fig.1). The dispersal kernels of species with greater IWLs had longer tails with wider 95% confidence intervals (shown clearly in Figure S1), as expected given the important effects of IWL and its interaction with maximum wind speed.

**Table 2:** Parameter estimates from the bootstrapped LMM model fitting IWL, maximum wind speed, and their interaction to log-transformed fruit dispersal distance of the 13 species released

Parameter	Estimate	(95% C.I.)	Approximate *P*-value
Intercept	0.517	(0.377, 0.817)	0.001
Log (IWL + 1)	0.186	(0.0759, 0.237)	0.001
Log (Maximum wind speed + 1)	−0.036	(−0.231, 0.171)	0.816
Log (IWL + 1 ^*^ Maximum wind speed + 1)	0.191	(0.115, 0.259)	0.001
Residual error (Std. Dev.)	0.558	(0.530, 0.591)	
Species random effect (Std. Dev.)	0.154	(0.144, 0.235)	

## Discussion

We found significant differences in dispersal distance among dipterocarp species based on their fruit morphologies: larger wing areas relative to fruit mass (IWL) facilitate lateral dispersal. Wind speed amplified these effects by increasing seed dispersal for all winged species, but particularly those with high IWL. Seed dispersal for all species was highly localized, with only 10% of fruit dispersing beyond 10 m. Although our experimental results were generated during a period of comparatively low wind speed, variation in wind speed during the course of the experimental releases allows us to build models from which dispersal distances at higher wind speed might be projected. We discuss our model results in relation to the theoretically predicted dispersal distances from mechanistic ballistic models and previous dipterocarp seed dispersal studies. Further, we highlight potential applications of the seed dispersal kernels generated using the LMM model.

The theoretical mechanisms linking wing loading measurements to dispersal distance are well established (Nathan et al. 2011), and experimental tests confirm that dispersal distances increase with both increasing wing area to mass ratio and increasing wind speed (Green 1980; Augspurger 1986; Augspurger and Franson 1987; Greene and Johnson 1989). The mean dispersal distances predicted using the LMM developed in this study (Table3) are lower than those predicted by the simple ballistic model of Nathan et al. (2011), as used in previous studies (Green 1980; Augspurger 1986; Matlack 1987). This in itself is not surprising, as the ballistic model applies a constant lateral wind speed throughout each fruits’ fall. While this might be applicable to seed dispersal in an open landscape (Nathan et al. 2008), it does not reflect wind speed variation within a tropical forest where a rapid decline in wind speed is encountered with increasing vertical displacement beneath the forest canopy (Aoki et al. 1975; Whitmore 1998). The ballistic model estimates are hence idealistic, inappropriate for forest conditions, and therefore likely to overestimate dispersal distances in tropical rain forest settings. More recent mechanistic models of wind dispersal do incorporate complex wind dynamics and turbulence (Kuparinen et al. 2007; Nathan et al. 2011; Fontan et al. 2013; Damschen et al. 2014), but these require accurate and continuous measures of wind speed along multiple axes and heights within the canopy and are hence generally restricted to computer simulations or laboratory experiments. These data were not available in this study, and obtaining such information is neither practical nor relevant for the purpose of generating generic dispersal models. Nonetheless, we are able to differentiate seed dispersal kernels among species based on fruit morphologies.

**Table 3:** Comparison of median predicted dispersal distances (m) from the bootstrapped LMM model presented in this study and distance predicted from the ballistic model when released from a height of 30 m and wind speed of 1.72 m/sec

Species	Mean IWL (cm^2^/g)	(Wing loading)^1/2^*	Predicted distance dispersed (m)	
			LMM model	Ballistic model†
*Dipterocarpus humeratus*	9.29 (± 0.27)	326.37	4.25	21.93
*Dipterocarpus kerrii*	8.93 (± 0.47)	346.34	3.90	20.54
*Hopea beccariana*	26.39 (± 0.83)	189.54	5.30	40.45
*Shorea acuminatissima*	17.37 (± 0.75)	231.89	4.13	32.07
*Shorea argentifolia*	44.60 (± 1.86)	147.71	7.90	54.53
*Shorea beccariana*	21.55 (± 0.45)	207.50	5.86	36.42
*Shorea falciferoides*	24.85 (± 1.03)	199.76	5.31	38.05
*Shorea gibbosa*	11.53 (± 0.58)	292.07	3.08	24.78
*Shorea macroptera*	39.83 (± 1.98)	162.65	6.70	48.50
*Shorea mexistopteryx*	16.49 (± 0.73)	242.07	5.48	30.55
*Shorea seminis*	1.97 (± 0.14)	724.03	2.27	9.41
*Shorea smithiana*	20.95 (± 0.65)	212.99	5.18	35.34
*Shorea xanthophylla*	0.00 (± 0.00)	0.00	1.76	NA

* Mean IWL (cm^2^/g) was converted to (wing loading)^1/2^ (in unit millidynes cm^2^) by first converting fruit mass (g) to millidynes and dividing by wing area (cm^2^), before square-rooting this value.

† The rate of descent *V*_*t*_ per species was calculated from the regression fitted values from “helicopter” fruit class from Table3 in Augspurger (1986).

Our results corroborate experimental and observational studies in concluding that seed dispersal in this family is predominantly local (Whitmore and Burnham 1984; Ashton 2004). Under similar wind conditions to this study (mean and maximum wind speed of 0.65 and 1.93 m/sec), the mean dispersal distances of *Dipterocarpus crinitus* and *Dipterocarpus cornutus* fruits (IWL 10.62 and 7.91, respectively) released from a 40-m canopy tower were 9 and 7 m, respectively (Osada et al. 2001). Itoh et al. (1997) observed the greatest density of newly established seedlings within 10 m of the mother tree for *Dryobalanops lanceolata* and *Dryobalanops aromatica*, with none found beyond 40 m. Fox (1972) observed a rapid decrease in fruit dispersing from 10 to 40 m across 12 dipterocarp species from four genera (*Dipterocarpus*, *Dryobalanops*, *Parashorea,* and *Shorea*), with on average only 9.1% of fruit reaching 40 m. Similarly, data compiled by Tamari and Jacalne (1984) from 12 species from the same four genera (three species overlapping with Fox) recorded maximum dispersal distances ranging 20 to 80 m, with the majority of seed dispersing 20–40 m. Even more extreme, wingless fruits or those possessing rudimentary but ineffective wings precluding gyration (30% of dipterocarps) (Suzuki and Ashton 1996) do not disperse beyond the crown of the mother tree; for example, 98% of *Shorea fallax* fruit fell within 10 m (Whitmore and Burnham 1984). Ridley’s premise (1930) that, barring extreme events, dipterocarp species attain maximum fruit dispersal distances of 100 yards (90 m) appears sound. Nonetheless, it is the extreme events that might have disproportionate ecological importance (Nathan 2006; Nathan et al. 2008).

The maximum observed dispersal distance of 39.54 m in this study, by a single *Shorea argentifolia* seed, is 50% of the maximum observed by Tamari and Jacalne (1984) and short of Ridley’s 90 m (1930). This suggests that while our study models short distance dispersal, the experimental release of fruit did not reflect the full range of natural dispersal events. This is likely due to the normal atmospheric conditions and hence relatively low wind speeds under which the experiment was conducted. However, the release height of 30 m is also slightly lower than the maximum heights of the study species of 40–60 m (Ashton 2004). Our models also take no account of wind turbulence (Tackenberg 2003; Bohrer et al. 2008). Long distance dispersal of wind-dispersed seed is primarily expected to occur with unusual or extreme atmospheric conditions, particularly those which cause strong updrafts (Tackenberg 2003; Wright et al. 2008). Fruits might also be disproportionately released during periods of high wind speed or persistent updrafts, such as those preceding storm events (Soons and Bullock 2008; Greene and Quesada 2011; Maurer et al. 2013). As Ridley (1930) acknowledges, this is pertinent to dipterocarp dispersal, with anecdotal reports of fruit being dispersed many 100s of meters by strong updrafts (Webber 1934; Whitmore and Burnham 1984). The frequency of such events, the dispersal distances attained, and subsequent fate of these seeds remain unknown. Nevertheless, the positive interaction effect among wind speed and IWL observed in our model implies that species with high IWL might disproportionately extend their seed dispersal range during high winds (Figure S1). IWL might therefore serve as a simple, albeit crude, proxy for LDD in this system, allowing conservationists and forest managers to identify dipterocarp species that might be most reproductively vulnerable to habitat fragmentation.

Our results have allowed us to develop a generalized dispersal model for dipterocarp species based on only two variables, IWL and wind speed. Using this model, we provide projected seed dispersal kernels for all dipterocarp species with IWLs of 1 to 50, representing 75% of those found in the region (Table S2). We additionally provide the parameter estimates for our bootstrapped LMM model (Table S1) for simulating dispersal kernels of any dipterocarp species. We believe that this model provides a robust basis for estimating dispersal kernels across the family under a range of typical wind conditions. This model can be extended to higher wind speeds, but this requires further experimental validation. This model has utility for projecting species’ dispersal patterns, information that is particularly relevant in the context of degraded, logged, and fragmented forests where patterns of gap formation and distribution might be very different to that of undisturbed forests. Furthermore, variation in IWL provides a theoretical framework to guide trait-based analyses of dipterocarp ecology (McGill et al. 2006; Westoby and Wright 2006), including trade-offs in reproductive traits (Westoby et al. 1996), demographic rates (Poorter et al. 2008), and community assembly.

## Supporting Information

**Data S1.** Generating dispersal kernels and calculation of approximate *P*-values

**Data S2.** R script of the analysis performed.

**Table S1.** Parameter estimates from the bootstrapped LMM model fitting IWL, maximum wind speed and their interaction to log-transformed fruit dispersal distance using the “new species” approach for unidentified dipterocarps.

**Table S2.** Median and 95% C.I. percentile dispersal distances predicted using the “new species” approach for dipterocarp species with IWLs 1 to 50 and wind speeds 1 to 10 m/s.

**Figure S1.** Simulated dispersal kernels of *S. seminis*, *S. smithiana* and *S. argentifolia* at maximum wind speed 1 and 5 m/sec (dashed and full lines respectively) with associated 95% confidence bands.

## References

[b1] Aoki M, Yabuki K, Koyama H (1975). Micrometeorology and assessment of primary productivity of a tropical rain forest in West Malaysia. J. Agric. Meteorol.

[b2] Ashton PS, Soedadmo E, Saw LG, Chung RCK (2004). Dipterocarpaceae. Tree flora of Sabah and Sarawak.

[b3] Augspurger CK (1986). Morphology and dispersal potential of wind-dispersed diaspore of neotropical trees. Am. J. Bot.

[b4] Augspurger CK, Franson SE (1987). Wind dispersal of artificial fruits varying in mass, area and morphology. Ecology.

[b5] Bagchi R, Henrys PA, Brown PE, Burslem DFRP, Diggle PJ, Gunatilleke CVS (2011). Spatial patterns reveal negative density dependence and habitat associations in tropical trees. Ecology.

[b100] Bates D, Maechler M, Bolker B, Walker S (2013). http://CRAN.R-project.org/package=lme4.

[b6] Bohrer G, Katul GG, Nathan R, Walko RL, Avissar R (2008). Effects of canopy heterogeneity, seed abscission and inertia on wind-driven dispersal kernels of tree seeds. J. Ecol.

[b7] Chesson P (2000). Mechanisms of maintenance of species diversity. Annu. Rev. Ecol. Syst.

[b8] Connell JH, Den Boer PJ, Gradwell GR (1971). On the role of natural enemies in preventing competitive exclusion in some marine animals and in rain forest tress. Dynamics of numbers populations.

[b9] Curran LM, Leighton M (2000). Vertebrate responses to spatiotemporal variation in seed production of mast-fruiting dipterocarpaceae. Ecol. Monogr.

[b10] Damschen EI, Baker DV, Bohrer G, Nathan R, Orrock JL, Turner JR (2014). How fragmentation and corridors affect wind dynamics and seed dispersal in open habitats. Proc. Natl Acad. Sci.

[b11] Davies S, Tan S, LaFrankie J, Roubik D, Sakai S, Hamid Karim A, Potts M (2005). Soil-related floristic variation in a hyperdiverse dipterocarp forest. Pollination ecology and the rain forest.

[b12] Fisher B, Edwards DP, Giam XL, Wilcove DS (2011). The high costs of conserving Southeast Asia’s lowland rainforests. Front. Ecol. Environ.

[b13] Fontan S, Katul GG, Poggi D, Manes C, Ridolfi L (2013). Flume experiments on turbulent flows across gaps of permeable and impermeable boundaries. Bound.-Layer Meteorol.

[b14] Fox JED (1972). The natural vegetation of Sabah and natural regeneration of the dipterocarp forest.

[b15] Fox JED (1973). Kabili-Sepilok forest reserve.

[b16] Gelman A, Hill J (2007). Data analysis using regression and multilevel/hierarchical models.

[b17] Green DS (1980). The terminal velocity and dispersal of spinning samaras. Am. J. Bot.

[b18] Greene DF, Johnson EA (1989). A model of wind dispersal of winged or plumed seeds. Ecology.

[b19] Greene DF, Quesada M (2011). The differential effect of updrafts, downdrafts and horizontal winds on the seed abscission of *Tragopogon dubius*. Funct. Ecol.

[b20] Hanski I, Zurita GA, Bellocq MI, Rybicki J (2013). Species-fragmented area relationship. Proc. Natl Acad. Sci. USA.

[b21] Hubbell SP (2001). The unified neutral theory of biodiversity and biogeography.

[b22] Itoh A, Yamakura T, Ogino K, Lee HS, Ashton PS (1997). Spatial distribution patterns of two predominant emergent trees in a tropical rainforest in Sarawak, Malaysia. Plant Ecol.

[b23] Janzen DH (1970). Herbivores and the number of tree species in tropical forests. Am. Nat.

[b24] Kuparinen A, Markkanen T, Riikonen H, Vesala T (2007). Modeling air-mediated dispersal of spores, pollen and seeds in forested areas. Ecol. Model.

[b25] Matlack GR (1987). Size, shape, and fall behaviour in wind-dispersed plant species. Am. J. Bot.

[b26] Maurer KD, Bohrer G, Medvigy D, Wright SJ, Thompson K (2013). The timing of abscission affects dispersal distance in a wind-dispersed tropical tree. Funct. Ecol.

[b27] Maycock CR, Thewlis RN, Ghazoul J, Nilus R, Burslem DFRP (2005). Reproduction of dipterocarps during low intensity masting events in a Bornean rain forest. J. Veg. Sci.

[b28] McGill BJ, Enquist BJ, Weiher E, Westoby M (2006). Rebuilding community ecology from functional traits. Trends Ecol. Evol.

[b29] Nathan R (2006). Long-distance dispersal of plants. Science.

[b30] Nathan R, Schurr FM, Spiegel O, Steinitz O, Trakhtenbrot A, Tsoar A (2008). Mechanisms of long-distance seed dispersal. Trends Ecol. Evol.

[b31] Nathan R, Katul G, Bohrer G, Kuparinen A, Soons M, Thompson S (2011). Mechanistic models of seed dispersal by wind. Theor. Ecol.

[b32] Newbery DM, Campbell EJF, Lee YF, Ridsdale CE, Still MJ (1992). Primary lowland dipterocarp forest at Danum Valley, Sabah, Malaysia: structure, relative abundance and family composition. Philos. Trans. R. Soc. Lond. B Biol. Sci.

[b33] Newman MF, Burgess PF, Whitmore TC (1996). Borneo island light hardwoods. Manuals of dipterocarps for foresters.

[b34] Newman MF, Burgess PF, Whitmore TC (1998). Borneo island medium and heavy hardwoods. Manuals of dipterocarps for foresters.

[b35] Osada N, Takeda H, Furukawa A, Awang M (2001). Fruit dispersal of two dipterocarp species in a Malaysian rain forest. J. Trop. Ecol.

[b200] Pinheiro J, Bates D, DebRoy S, Sarkar D, R Core Team (2013). http://CRAN.R-project.org/package=nlme.

[b36] Poorter L, Wright SJ, Paz H, Ackerly DD, Condit R, Ibarra-Manríquez G (2008). Are functional traits good predictors of demographic rates? Evidence from five Neotropical forests. Ecology.

[b300] R Core Team (2013). http://www.R-project.org.

[b37] Rees M, Condit R, Crawley M, Pacala S, Tilman D (2001). Long-term studies of vegetation dynamics. Science.

[b38] Ridley HN (1930). The dispersal of plants throughout the world.

[b39] Seidler TG, Plotkin JB (2006). Seed dispersal and spatial pattern in tropical trees. PLoS Biol.

[b40] Sodhi NS, Posa MRC, Lee TM, Bickford D, Koh LP, Brook BW (2009). The state and conservation of Southeast Asian biodiversity. Biodivers. Conserv.

[b41] Soons MB, Bullock JM (2008). Non-random seed abscission, long-distance wind dispersal and plant migration rates. J. Ecol.

[b42] Suzuki E, Ashton PS (1996). Sepal and nut size ratio of fruits of Asian Dipterocarpaceae and its implications for dispersal. J. Trop. Ecol.

[b43] Tackenberg O (2003). Modeling long-distance dispersal of plant diaspores by wind. Ecol. Monogr.

[b44] Tamari C, Jacalne DV (1984). Fruit dispersal of dipterocarps. Bull. For. For. Prod. Res. Inst.

[b45] Tilman D (1994). Competition and biodiversity in spatially structured habitats. Ecology.

[b46] Tutiempo (2014). http://en.tutiempo.net/climate/ws-964910.html.

[b47] Webber ML (1934). Fruit dispersal. Malay. Forest.

[b48] Wells K, Bagchi R (2005). Eat in or take away - Seed predation and removal by rats (muridae) during a fruiting event in a dipterocarp rainforest. Raffles Bull. Zool.

[b49] Westoby M, Wright IJ (2006). Land-plant ecology on the basis of functional traits. Trends Ecol. Evol.

[b50] Westoby M, Leishman M, Lord J, Poorter H, Schoen DJ (1996). Comparative ecology of seed size and dispersal [and discussion]. Philos. Trans. Biol. Sci.

[b51] Whitmore TC (1998). An introduction to tropical rain forests.

[b52] Whitmore TC, Burnham CP (1984). Tropical rain forests of the Far East.

[b53] Wilcove DS, Giam X, Edwards DP, Fisher B, Koh LP (2013). Navjot’s nightmare revisited: logging, agriculture, and biodiversity in Southeast Asia. Trends Ecol. Evol.

[b54] Wright SJ, Trakhtenbrot A, Bohrer G, Detto M, Katul GG, Horvitz N (2008). Understanding strategies for seed dispersal by wind under contrasting atmospheric conditions. Proc. Natl Acad. Sci.

