# Supplementary material for: Predicting dispersal of auto-gyrating fruit in tropical trees: a case study from the Dipterocarpaceae
Source: Ecol Evol. 2015 Apr 2;5(9):1794–801. doi: 10.1002/ece3.1469 (PMC4485961; doi:10.1002/ece3.1469)
Supplement: Supplementary file 1 — Data S1. Generating dispersal kernels and calculation of approximate P-values [file ece30005-1794-sd1.docx]

**Supplement S1.**

**Generating dispersal kernels and calculation of approximate *p*-values**

Dispersal kernels and their confidence intervals were estimated using a parametric bootstrapping approach implemented with the “bootMer” function in lme4. This function generates a new response by simulating a set of residuals drawn from a normal distribution of mean 0 and variance equal to the residual variance of the model, and adding this to the model fitted values. In addition, the random effects were dealt with in two ways: (1) the best linear unbiased predictor (BLUP) ([Pinheiro and Bates 2000](#_ENREF_28)) of the difference of each species’ mean dispersal distance to the expectation based on the fixed effects was added to the simulated responses for that species; or (2) new BLUPS were drawn for each species from a normal distribution of mean 0 and variance equal to the variance component for species from the model. The first approach (hereafter the “known species” approach) corresponds to the situation where we are simulating dispersal for the set of species used to fit the original model. In the second approach (hereafter “new species” approach), the simulated data represents a new set of species drawn from the same distribution as the original set. We ran 1000 simulations under both approaches and then refitted the model to these simulated data. This resulted in 1000 simulated models from both approaches.

Dispersal kernels for each species, and their confidence bands, were estimated from the prediction intervals of the simulated “known species” models given the mean IWL for the species and setting maximum wind speed to the mean observed during the experiment (1.72 m/s). We used each of the 1000 models to simulate 1000 dispersal distances for each species. These distances were simulated by summing the model’s expectation for the species and a vector of 1000 random numbers drawn from a normal distribution with mean 0 and variance equal to the model’s residual variance. We back-transformed the distances to the original scale (by taking the exponent) and then extracted the percentiles of these distances between 1 and 99 with increments of 1. We calculated the median distance for each percentile across the 1000 simulated “known species” models. This value represents the distance to which the corresponding percentage of seeds disperses. Confidence bands were similarly estimated as the 0.25 and 0.975 quantiles across the 1000 models for each percentile. Dispersal kernels for a sample of 50 hypothetical dipterocarp species with IWLs spanning 1 to 50 were simulated in a similar method to above; however the models from the “new species” approach were used as species identities were not known.

The calculation of *p*-values, and hence testing of hypotheses using LMM is much debated and no consensus methodology has emerged. The bootstrap approach allows us to circumvent these problems by calculating approximate, two-tailed *p*-values from the posterior distribution of the 1000 models as recommended by Gelman & Hill ([2007](#_ENREF_11)) using the formula:

$p=1-2 \left| \frac{x}{1000}-0.5 \right|$,

where *x* equals the number of samples greater than zero.
