# Supplementary material for: Predicting dispersal of auto-gyrating fruit in tropical trees: a case study from the Dipterocarpaceae
Source: Ecol Evol. 2015 Apr 2;5(9):1794–801. doi: 10.1002/ece3.1469 (PMC4485961; doi:10.1002/ece3.1469)
Supplement: Supplementary file 3 — Table S1. Parameter estimates from the bootstrapped LMM model fitting IWL, maximum wind speed and their interaction to log-transformed fruit dispersal distance using the “new species” approach for unidentified dipterocarps. [file ece30005-1794-sd3.docx]

**Supplement S2**

**Table S1.** Parameter estimates from the bootstrapped LMM model fitting IWL, maximum wind speed and their interaction to log-transformed fruit dispersal distance using the “new species” approach for unidentified dipterocarps.

| **Parameter** | **Estimate** | **95% C.I.** | **Approximate *p*-value** |  |
| --- | --- | --- | --- | --- |
| Intercept | 0.559 | (0.269, 0.838) | 0.001 |  |
| Log(IWL + 1) | 0.170 | (0.071, 0.274) | 0.001 |  |
| Log(Maximum wind speed + 1) | -0.0353 | (-0.244, 0.173) | 0.802 |  |
| Log(IWL +1 * Maximum wind speed + 1) | 0.189 | (0.116, 0.264) | 0.001 |  |
| Residual error (st. Dev.) | 0.558 | (0.531, 0.588) |  |  |
| Species random effect (st. Dev) | 0.152 | (0.106, 0.274) |  |  |
