# Supplementary material for: Predicting dispersal of auto-gyrating fruit in tropical trees: a case study from the Dipterocarpaceae
Source: Ecol Evol. 2015 Apr 2;5(9):1794–801. doi: 10.1002/ece3.1469 (PMC4485961; doi:10.1002/ece3.1469)
Supplement: Supplementary file 5 — Figure S1. Simulated dispersal kernels of S. seminis, S. smithiana and S. argentifolia at maximum wind speed 1 and 5 m/sec (dashed and full lines respectively) with associated 95% confidence bands. [file ece30005-1794-sd5.docx]

**Supplement S3**


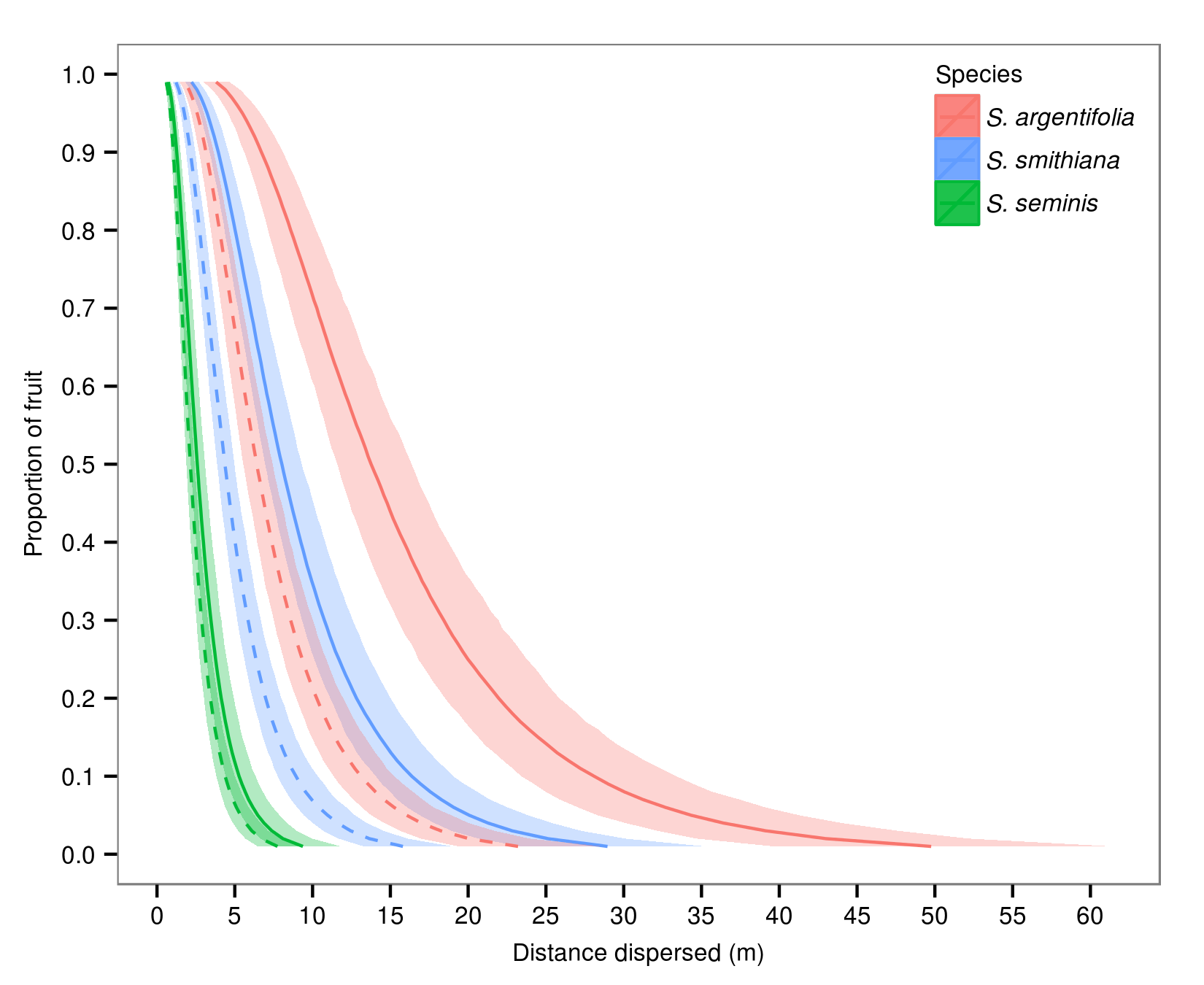


**Figure S1.** Simulated dispersal kernels of *S. seminis*, *S. smithiana* and *S. argentifolia* at maximum wind speed 1 and 5 m/s (dashed and full lines respectively) with associated 95% confidence bands.
